# Activation of the inflammasome and pyroptosis cascade in podocytes of patients with minimal change disease

**DOI:** 10.1093/ckj/sfae216

**Published:** 2024-07-16

**Authors:** Yuki Kajio, Taihei Suzuki, Kazuki Kobayashi, Nobuhiro Kanazawa, Masayuki Iyoda, Hirokazu Honda, Kazuho Honda

**Affiliations:** Division of Nephrology, Department of Medicine, Showa University School of Medicine, Tokyo, Japan; Department of Anatomy, Showa University School of Medicine, Tokyo, Japan; Division of Nephrology, Department of Medicine, Showa University School of Medicine, Tokyo, Japan; Division of Nephrology, Department of Medicine, Showa University School of Medicine, Tokyo, Japan; Division of Nephrology, Department of Medicine, Showa University School of Medicine, Tokyo, Japan; Division of Nephrology, Department of Medicine, Showa University School of Medicine, Tokyo, Japan; Department of Microbiology and Immunology, Showa University School of Medicine, Tokyo, Japan; Division of Nephrology, Department of Medicine, Showa University School of Medicine, Tokyo, Japan; Department of Anatomy, Showa University School of Medicine, Tokyo, Japan

**Keywords:** CD36, inflammasome, minimal change disease, nephrotic syndrome, pyroptosis

## Abstract

**Background:**

In contrast to childhood minimal change disease (MCD), adult-onset MCD frequently recurs and requires prolonged immunosuppressive therapy. Accordingly, an investigation of the pathogenesis of adult MCD is required. MCD is usually accompanied by severe dyslipidaemia. Oxidized low-density lipoprotein (ox-LDL) is known to function in a damage-associated molecular pattern (DAMP) through CD36, triggering the NOD-like receptor thermal protein domain-associated protein 3 (NLRP3) inflammasome and programmed cell death called pyroptosis. However, the relationship between MCD pathogenesis and NLRP3 inflammasome/pyroptosis activation via CD36 is not fully understood.

**Methods:**

We conducted comprehensive histological and clinical evaluations by analysing renal biopsy (RBx) specimens and urine samples obtained from 26 patients with MCD. These samples were compared with control kidneys from 15 transplant donors and urine samples from 15 healthy volunteers.

**Results:**

The number of podocytes was lower in the MCD group than in the control group. Urinary ox-LDL levels were higher in the MCD group than in the control group. Immunofluorescence staining revealed that NLRP3 and CD36 were upregulated in MCD podocytes. Urinary interleukin (IL)-18 levels increased in patients with MCD. Steroid therapy performed before RBx appeared to maintain the podocyte number and reduce urinary ox-LDL and IL-18 levels.

**Conclusion:**

In MCD, the NLRP3 inflammasome and pyroptosis cascade seem to be activated via upregulation of CD36 in podocytes, associated with increased urinary ox-LDL. Elevated urinary IL-18 levels suggest that pyroptosis may occur in MCD. Further research is required to confirm the significance of the podocyte NLRP3 inflammasome/pyroptosis in MCD.

## Graphical Abstract

**Graphical Abstract:**
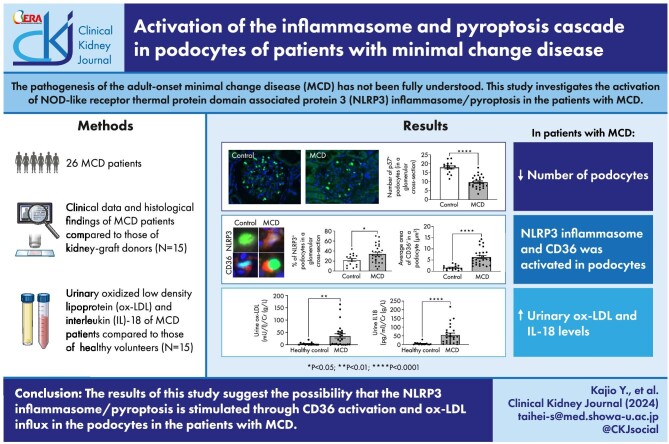

KEY LEARNING POINTS **What was known:**

- Adult-onset minimal change disease (MCD) frequently reoccurs and requires prolonged immunosuppressive therapy compared with childhood MCD; thus, an investigation of the pathogenesis of adult MCD is required.
- Severe dyslipidaemia is frequently present in patients with MCD.
- Oxidized low-density lipoprotein (ox-LDL) activates the NOD-like receptor thermal protein domain-associated protein 3 (NLRP3) inflammasome and induces programmed cell death, called pyroptosis, through CD36.

**This study adds:**

- In patients with MCD, the number of podocytes was decreased when compared with that in the control group, indirectly indicating that podocyte death could be associated with MCD pathogenesis.
- Urinary ox-LDL levels increased, and CD36 upregulation and NLRP3 activation occurred in MCD podocytes, suggesting that pyroptosis may be induced in MCD.
- Steroid therapy did not seem to contribute to the regulation of NLRP3 and pyroptosis cascade.

**Potential impact:**

- Targeting the regulation of the NLRP3 inflammasome and pyroptosis cascade could be a therapeutic strategy against adult-onset MCD.

## INTRODUCTION

Minimal change disease (MCD) serves as a prototypical nephrotic syndrome (NS), marked by the abrupt emergence of pronounced proteinuria, hypoalbuminemia and dyslipidaemia [1]. In general, MCD responds well to immunosuppressive therapy but frequently relapses. Adults with MCD experience a higher frequency of recurrence compared with children with MCD [2]. Therefore, prolonged steroid therapy is required for adult MCD patients [3–5]. Accordingly, we aimed to unveil the intricate pathogenesis of MCD and identify effective therapeutic strategies, particularly imperative for addressing adult-onset MCD. Electron microscopy findings in MCD revealed diffuse foot process effacement and podocyte degeneration. Consequently, podocyte injury emerged as pivotal in the pathogenesis of MCD. In the aftermath of severe injury, podocytes may undergo cell death programs, exacerbating NS. Nevertheless, a definitive investigation into the role of podocyte death in MCD remains outstanding.

Several cell death pathways exist, including apoptosis, necroptosis, ferroptosis and pyroptosis. Pyroptosis is characterized by the activation of the NOD-like receptor thermal protein domain associated protein 3 (NLRP3) inflammasome, leading to the release of pro-inflammatory cytokines, including interleukin (IL)-1β and IL-18 [6]. Activation of the NLRP3 inflammasome/pyroptosis is induced by various pathogen-associated molecular patterns and damage-associated molecular patterns (DAMPs) [7]. Oxidized low-density lipoprotein (ox-LDL) and cholesterol crystals are DAMPs involved in the NLRP3 inflammasome cascade. Severe dyslipidaemia is frequently accompanied by excessive hepatic protein production in the liver. Therapeutic benefits for NS through 3-hydroxy-3-methyl-glutaryl-coenzyme A reductase inhibitors and LDL-apheresis have been documented [8, 9]. However, the role of ox-LDL in the pathogenesis of MCD is not fully understood.

CD36 serves as a type B scavenger receptor for ox-LDL and fatty acids, and the influx of lipids through CD36 is believed to trigger the activation of the NLRP3 inflammasome/pyroptosis cascade [10]. This transmembrane protein is expressed in diverse cell types, including platelets, macrophages and endothelial cells [11], with predominant expression in podocytes [12]. This suggests that the activation of the NLRP3 inflammasome and pyroptosis could occur in MCD. In this study, we performed exploratory research focusing on the NLRP3 inflammasome and pyroptosis cascade in MCD using renal biopsy (RBx) and urine samples from patients with MCD.

## MATERIALS AND METHODS

### Ethical statement

This study was approved by the Ethics Committee of Showa University Hospital, Tokyo, Japan (No. 21-038-A), and patient data were used in accordance with the latest version of the Helsinki Declaration of Human Research Ethics.

### Patients

Fifty-six patients diagnosed with MCD, according to the pathological findings of RBx performed at Showa University Hospital (Tokyo, Japan) between April 2010 and March 2022, were included in this study. Informed consent was obtained for the study before RBx. Patients with defective clinical data and those without urine samples at the time of RBx were excluded. Ultimately, 26 patients were assessed. The clinical characteristics of patients with MCD analysed in this study were recorded at the time of hospitalization. Urine samples from patients with MCD were obtained during RBx. As a control, RBx samples from transplant donors (biopsied 0 h after kidney transplantation, *N* = 15) and serum and urine samples from healthy volunteers (*N* = 15) were used.

### Immunofluorescence staining

We performed double immunofluorescence staining for p57 with NLRP3, gasdermin D (GSDMD) or CD36 in paraffin-embedded sections. The paraffin-embedded materials were sectioned at 2-μm thickness. Deparaffinization was performed using xylene, and the sections were rehydrated in a graded ethanol series. Antigen retrieval was applied to deparaffinized sections in boiled 10 mM citric acid buffer (pH 6.0) at 94°C for 30 min. Non-specific protein reactions were blocked using a Background Buster (Innovex Bioscience Inc., Richmond, CA, USA) for 15 min at room temperature. Antibodies were diluted with 1% immunoglobulin G (IgG)-free bovine serum albumin in phosphate-buffered saline. After the blocking process, the sections were incubated with mouse anti-p57 antibody (1:600, Santacruz, Dallas, TX, USA) at 4°C overnight, followed by the application of anti-mouse IgG conjugated to Alexa Flour 488 (1:200, Thermo Fisher Scientific, Waltham, MA, USA) for 1 h at room temperature. Next, the sections were incubated with the antibodies for CD36 (1:100, Abcam, Cambridge, UK), NLRP3 (1:100, Abcam) and GSDMD (1:100, Proteintech, Rosemont, IL, USA) at 4°C overnight, followed by the application of goat anti-rabbit IgG antibody conjugated to Alexa Fluor 594 (1:200, Thermo Fisher Scientific) at room temperature for 1 h. All immunofluorescence-stained sections were mounted in Vectashield mounting medium with 4′, 6-diamidino-2-phenylindole (DAPI) (Vector Laboratories, Newark, CA, USA). For the double immunofluorescence staining of GSDMD and NLRP3, we used an additional antibody for GSDMD (mouse anti-human GSDMD, 1:50, Proteintech). Five glomeruli were selected for a blind assessment.

### Immunohistochemistry staining for IL-18

Immunohistochemistry staining for IL-18 was performed as described previously [13]. Briefly, paraffin sections (2 μm each) were used for immunohistochemical analysis of the IL-18^+^ area in the glomeruli. After deparaffinization, sections were fixed with acetone for 20 min at –20°C. After inactivating endogenous peroxidase by incubation with 0.3% hydrogen peroxide in methanol for 30 min at room temperature and blocking non-specific protein binding using Background Buster for 15 min at room temperature, the sections were incubated with the rabbit antibody against human IL-18 (1:100, Abcam) overnight at 4°C. The sections were then incubated with goat anti-rabbit IgG (1:1000, Abcam) for 1 h at room temperature and stained using a 3,3′ Diaminobenzidine chromogen/substrate kit (Agilent, Santa Clara, CA, USA). The nuclei were stained with haematoxylin. After dehydration with 95% and 100% ethanol and xylene, the sections were covered with a mounting medium.

### Assessment of immunofluorescence staining images

Images were captured using BZ-X800 (Keyence Corporation). Double immunofluorescence staining for p57 was used to analyse the number of podocytes in the glomerular cross-section. The average number of podocytes in the glomerular cross-section was quantified by counting the number of p57^+^ cells. Double immunofluorescence staining for p57 and NLRP3 was used to assess the percentage of NLRP3^+^ podocytes [(number of p57^+^ and NLRP3^+^/number of p57^+^ cells) × 100/glomerular cross-section]. Double immunofluorescence staining for p57 and CD36 was used to analyse the average CD36^+^ area (μm^2^) in a podocyte (p57^+^), which was assessed for each glomerular cross-section. The CD36^+^ area was analysed using ImageJ Fiji software (version 2.1.0/1.53c) [14].

### Enzyme-linked immunosorbent assay for urine ox-LDL and IL-18

Urinary ox-LDL (CUSABIO, Houston, TX, USA) and IL-18 (R&D Systems, Minneapolis, MN, USA) levels in patients with MCD (*N* = 26) and healthy volunteers (*N* = 15) were measured using a commercially available enzyme-linked immunosorbent assay (ELISA) kit, according to the manufacturer's instructions. Both ox-LDL (mU/L) and IL-18 (pg/mL) levels were normalized to the urinary creatinine (Cr) (g/L) level of each patient or a healthy volunteer.

### Statistical analysis

The data are expressed as the mean ± standard error of the mean (SEM). Statistical analysis of differences between the two groups was performed using the Mann–Whitney test. Statistical significance was set at a *P-*value of <.05. All statistical analyses were performed using GraphPad Prism version 10 (GraphPad Software).

## RESULTS

### Clinical characteristics of the patients enrolled in this study

The clinical characteristics of the kidney donors (treated as controls, *N* = 15) and patients with MCD (*N* = 26) are shown in Table 1. Age, serum Cr, total protein, albumin, LDL and triglyceride levels differed significantly between the control and MCD groups. Although serum Cr levels were higher in the MCD group, the estimated glomerular filtration rate was not statistically different between the groups.

**Table 1:** Clinical characteristics.

	Control (transplant donor, *N* = 15)	MCD (*N* = 26)	*P*-value
Age, years	61.53 ± 9.85	47.7 ± 16.23	.0042
Gender (female)	4 (11)	15 (11)	.10
DM [% (*N*)]	6.66 (1)	0 (0)	.36
HTN [% (*N*)]	20 (3)	23.33 (7)	>.99
DL [% (*N*)]	20 (3)	16.67 (5)	>.100
Serum Cr (mg/dL)	0.69 ± 0.11	0.95 ± 0.41	.015
eGFR (mL/min/1.73 m^2^)	72.62 ± 11.65	70.90 ± 24.70	.69
Serum TP	7.06 ± 0.24	4.32 ± 0.68	<.0001
Serum Alb	4.3 ± 0.18	1.32 ± 0.52	<.0001
Serum LDL (mg/dL)	124.53 ± 32.33	378.73 ± 145.94	<.0001
Serum HDL (mg/dL)	75.50 ± 27.59	82.15 ± 31.20	.63
Serum TG (mg/dL)	97.80 ± 54.57	274.07 ± 166.92	<.0001
Proteinurea (g/day)	N/A	8.74 ± 5.08	N/A

Data are presented as mean ± SEM or as % (*N*).

Alb, albumin; DL, dyslipidemia; DM, diabetes mellitus; eGFR, estimated glomerular filtration rate; HDL, high-density lipoprotein; HTN, hypertension; TP, total protein; TG, triglyceride.

### Number of podocytes in patients with MCD

**Figure 1:**
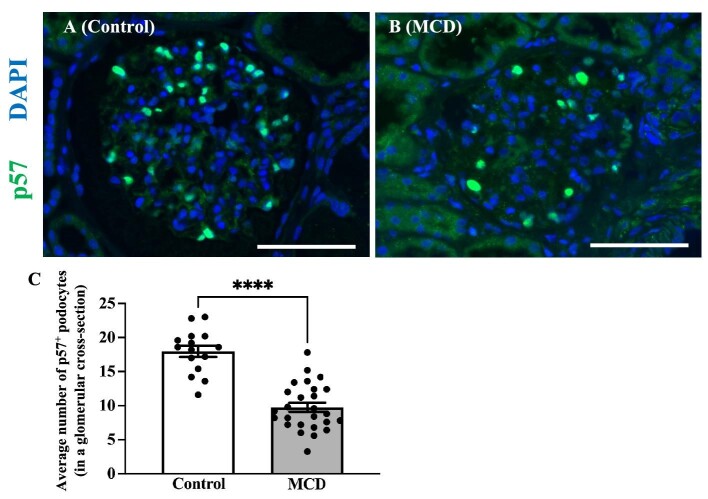
Reduced podocyte count revealed through p57 immunostaining in MCD compared with the control group. Representative images of immunofluorescence staining for p57 (green) and DAPI (blue) in control kidney donors (*N* = 15) (**A**) and MCD patients (*N* = 26) (**B**). Quantification of podocyte numbers in glomerular cross-sections (**C**). Magnification: ×400. Bar: 100 μm. Data are presented as the mean ± SEM. ^***^P < .001.

Immunofluorescent staining for p57 showed that the average number of p57^+^ podocytes in the glomerular cross-section was significantly lower in the patients with MCD compared with the kidney donors (9.75 ± 0.66 vs 17.96 ± 0.83, *P *< .001) (Fig. 1)

### Levels of urinary ox-LDL in patients with MCD

**Figure 2:**
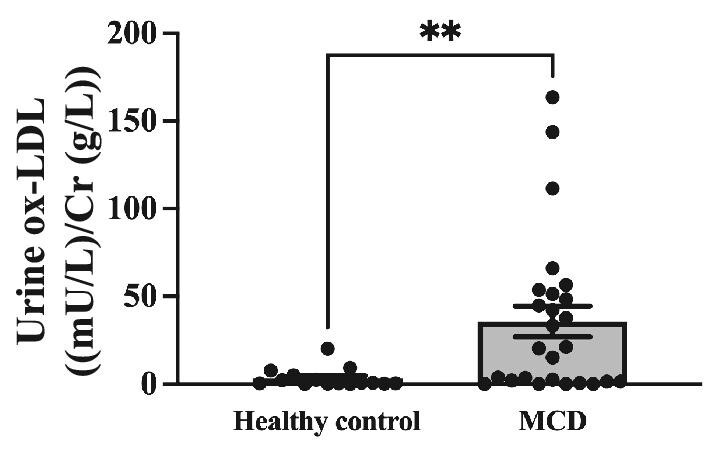
Elevated urinary ox-LDL levels observed in MCD compared with the control group. Urinary ox-LDL levels [mU/L/Cr (g/L)] were assessed in the control (*N* = 15) and MCD (*N* = 26) groups using ELISA. Data are presented as the mean ± SEM. ^**^P < .01.

We assessed the levels of urine ox-LDL using ELISA. Ox-LDL levels were normalized to the urine Cr levels. Compared with the control, urinary ox-LDL levels were significantly higher in the patients with MCD [34.61 ± 8.75 vs 3.36 ± 1.41, *P *< .01, mU/L/Cr (g/L)] (Fig. 2).

### NLRP3 inflammasome/pyroptosis in patients with MCD

**Figure 3:**
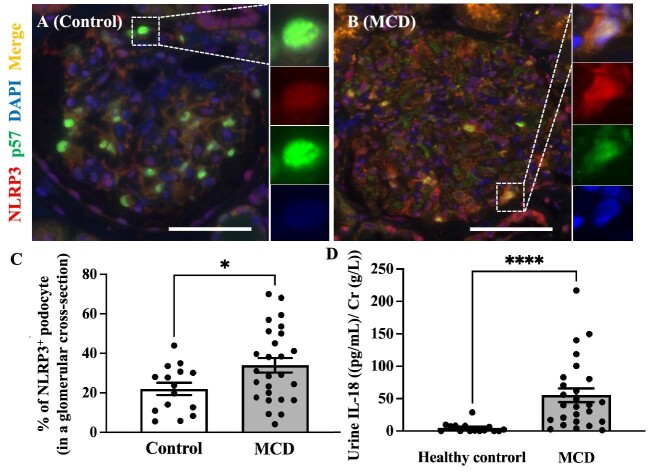
Activation of NLRP3/pyroptosis cascade in podocytes within MCD. Representative images of immunofluorescence staining for p57 (green), NLRP3 (red) and DAPI (blue) in control kidney donors (*N* = 15) (**A**) and patients with MCD (*N* = 26) (**B**). Quantification of the percentage of NLRP3^+^ podocytes in the glomerular cross-section (**C**). Urinary IL-18 levels [pg/mL/Cr (g/L)] were assessed using ELISA (**D**). Magnification: ×400. Bar: 100 μm. Dotted square: representative part of the image. Data are presented as the mean ± SEM. *P < .05; ^****^P < .0001.

**Figure 4:**
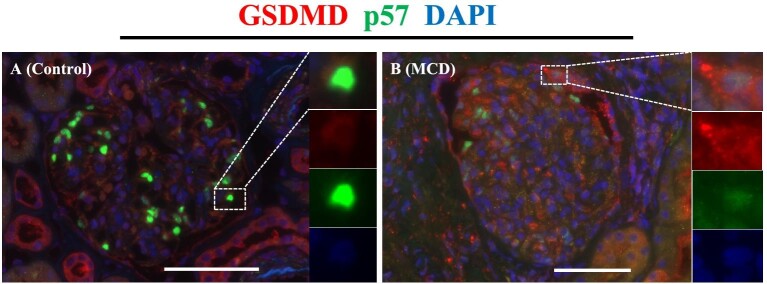
Immunofluorescence staining for p57 (green), GSDMD (red) and DAPI (blue) in the control and the MCD groups. Immunofluorescence staining for p57 and GSDMD showed that GSDMD^+^ podocytes were barely detectable in either control kidney donors or patients with MCD. Representative images of control (**A**) and MCD (**B**) groups. Magnification: ×400. Bar: 100 μm. Dotted square: representative part of the image.

**Figure 5:**
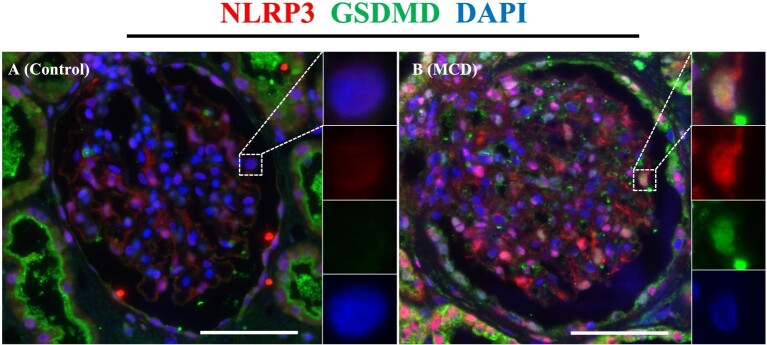
Immunofluorescence staining for GSDMD (green), NLRP3 (red) and DAPI (blue) in the control and the MCD group. Immunofluorescence staining for GSDMD^+^ podocytes also showed NLRP3 expression. Representative images of control (**A**) and MCD (**B**) groups. Magnification: ×400. Bar: 100 μm. Dotted square: representative part of the image.

We performed double immunofluorescent staining for NLRP3/p57 and GSDMD/p57 to reveal the activation of the NLRP3 inflammasome in the podocytes of patients with MCD. Compared with the control, the % of NLRP3^+^ podocyte in a glomerular cross-section was significantly increased in MCD (33.95 ± 3.65 vs 21.99 ± 3.08, *P *< .05) (Fig. 3C). Overview images are shown in Supplementary data, Fig. S1. In contrast, GSDMD^+^ podocytes were barely detectable in either group (Fig. 4). Additionally, we performed immunofluorescence staining for GSDMD and NLRP3, which showed that GSDMD^+^ podocytes also expressed NLRP3 in patients with MCD (Fig. 5).

**Figure 6:**
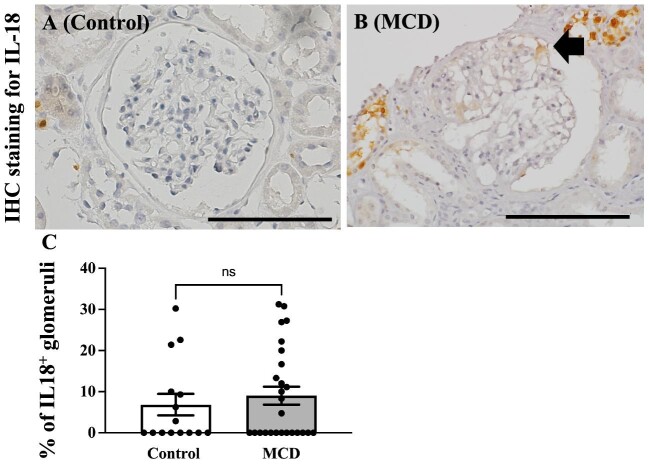
Immunohistochemistry staining for IL-18. Representative image of a glomerulus in the control and MCD groups. Immunohistochemical staining for IL-18 showed that IL-18^+^ cells were barely detectable in control kidney donors or patients with MCD. Representative images of control (**A**) and MCD (**B**) groups. Statistical analysis for the percentage of IL-18^+^ glomeruli was performed (**C**). Magnification: ×400. Bar: 100 μm. Arrow: IL-18^+^ cells are detected outside the glomerular tuft.

We conducted ELISA to compare urine IL-18 levels between the control and MCD groups, aiming to assess pyroptosis. The results revealed a significant elevation in urinary IL-18 levels among MCD patients compared with healthy controls [55.23 ± 10.46 vs 1.79 ± 1.94, *P* < .001, pg/mL/Cr (g/L)] (Fig. 3D). The predominant detection of IL-18 occurred in the tubules for both the control and MCD groups (data not shown), with only a minimal presence of IL-18^+^ podocytes in the glomerular tufts (Fig. 6). Overview images are shown in the Supplementary data, Fig. S2.

### CD36 expression of the podocytes in patients with MCD

**Figure 7:**
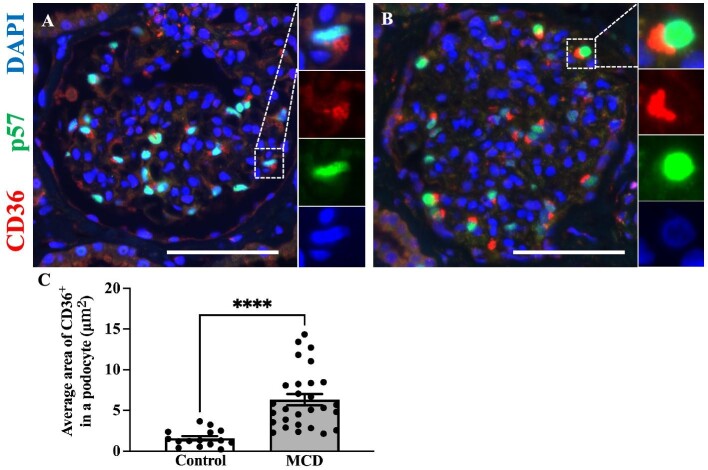
Increased CD36 expression in MCD podocytes. Representative images of double immunofluorescence staining for p57 (green), CD36 (red) and DAPI (blue) in controls (*N* = 15) (**A**) and MCD (*N* = 26) (**B**). Quantification of the CD36^+^ area (μm^2^) in podocytes (**C**). Magnification: ×400. Bar: 100 μm. Dotted square: representative part of the image. Data are presented as mean ± SEM. ^****^P < .0001.

Double immunofluorescence staining for CD36 and p57 was performed to determine differences in CD36 expression in podocytes between the control and MCD groups (Fig. 7A and B). The average CD36^+^ area in a podocyte was significantly larger in MCD compared with the control group (6.32 ± 0.67 vs 1.58 ± 0.26, *P *< .001, μm^2^) (Fig. 7C).

### Clinical characteristics of MCD patients before RBx

We divided the patients into two groups according to the application of steroid therapy before RBx and statistically compared the clinical characteristics between the groups [therapy (–) before RBx, *N* = 14; therapy (+) before RBx, *N* = 12] (Table 2). Serum Cr levels and estimated glomerular filtration rate were significantly different between the groups.

**Table 2:** Clinical characteristics of the MCD patients with our without steroid therapy.

	Therapy (–) before RBx (*N* = 14)	Therapy (+) before RBx (*N* = 12)	*P*-value
Age, years	48.86 ± 12.41	47.08 ± 17.90	.89
Gender (female)	7 (7)	8 (4)	.45
DM [% (*N*)]	0 (0)	0 (0)	N/A
HTN [% (*N*)]	4 (28.57)	2 (16.67)	.65
DL [% (*N*)]	4 (28.57)	0 (0)	.1
Serum Cr (mg/dL)	0.78 ± 0.26	1.20 ± 0.48	.0056
eGFR (mL/min/1.73 m^2^)	77.01 ± 21.19	60.15 ± 26.58	.046
Serum TP	4.59 ± 0.71	4.11 ± 0.48	.059
Serum Alb	1.50 ± 0.64	1.13 ± 0.21	.056
Serum LDL (mg/dL)	343.50 ± 141.75	424.83 ± 121.33	.069
Serum HDL (mg/dL)	89.39 ± 33.27	74.41 ± 26.13	.25
Serum TG (mg/dL)	256.0 ± 133.07	332.0 ± 197.93	.46
Proteinurea (g/day)	7.59 ± 4.24	10.31 ± 5.84	.19

Data are presented as mean ± SEM or as % (*N*).

Alb, albumin; DL, dyslipidemia; DM, diabetes mellitus; eGFR, estimated glomerular filtration rate; HDL, high-density lipoprotein; HTN, hypertension; TP, total protein; TG, triglyceride.

### Number of podocytes in MCD patients who received steroid therapy before RBx

Immunofluorescence staining for p57 revealed that the number of podocytes in a glomerular cross-section was maintained in the MCD patients who received steroid therapy (11.12 ± 0.97 vs 6.84 ± 1.18, *P *< .05) (Fig. 7A).

### Impact of steroid therapy on CD36, NLRP3, urinary IL-18 and ox-LDL levels in podocytes

**Figure 8:**
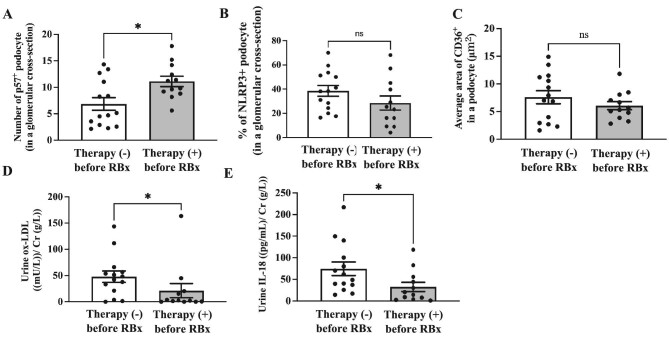
Comparative analysis of podocyte count, NLRP3 and CD36 expression in podocytes, and urinary ox-LDL and IL-18 levels between steroid therapy (–) and (+) groups prior to renal biopsy. Steroid therapy seems to contribute to maintaining the number of podocytes and reducing urinary ox-LDL and IL-18 levels in MCD, whereas NLRP3 and CD36 expression in podocytes is not affected by steroid use. Number of podocytes in the glomerular cross-section (**A**). The average area of CD36^+^ area in a podocyte (μm^2^) (**B**). Percentage of NLRP3^+^ podocytes in the glomerular cross-section (**C**). Urinary ox-LDL [mU/L/Cr (g/L)] (**D**) and IL-18 [pg/mL/Cr (g/L)] (**E**) levels were assessed using ELISA. Therapy (–), *N* = 14; therapy (+), *N* = 12. Data are presented as the mean ± SEM. *P < .05.

We assessed the expression of NLRP3 and CD36 in podocytes from patients who underwent steroid therapy, comparing them with those who did not, employing the same methodology as described earlier during RBx. There was no significant difference in the expression of NLRP3 (28.56 ± 5.81 vs 38.58 ± 4.43, *P *> .05, %) (Fig. 8B) and CD36 (6.07 ± 0.73 vs 7.59 ± 1.17, *P *> .05, μm^2^) (Fig. 8C) in the podocytes. On the contrary, steroid therapy before RBx significantly reduced the levels of urinary IL-18 [32.67 ± 10.65 vs 74.58 ± 15.69, *P *< .05, pg/mL/Cr (g/L)] (Fig. 8D) and ox-LDL [21.23 ± 13.50 vs 47.95 ± 10.79, *P *< .05, pg/mL/Cr (g/L)] (Fig. 8E).

## DISCUSSION

Podocyte injury, predominantly functional rather than lethal, is regarded as the primary pathogenic factor in MCD. The involvement of a cell death program in podocytes in the progression of MCD remains a subject of controversy. Although podocyte death is difficult to define and quantify, podocyte loss may be a surrogate marker for podocyte death. Typically, discussions about podocyte loss have predominantly centred around NS, encompassing conditions such as focal segmental glomerulosclerosis, in addition to MCD [15]. However, several reports have indicated that podocyte loss can be detected in patients with MCD, similar to other NS [16–18]. In addition, some studies have demonstrated an increase in the number of urinary podocytes in patients with MCD [19, 20]. These results indirectly suggest that podocyte detachment from the glomerular tufts occurs in MCD. Accordingly, we first assessed the podocyte number in the glomerular tuft using RBx staining for p57 in patients with MCD compared with kidney donors. In our study, the number of podocytes was significantly lower in the MCD group than in the control group. In addition, a greater number of podocytes was detected in patients with MCD who underwent steroid therapy than in those who did not use steroids at the time of RBx. These results suggest that podocyte death may be related to the pathogenesis of MCD, which can be prevented using steroids.

Various cell death pathways, encompassing apoptosis, necroptosis, ferroptosis and pyroptosis, are recognized. Apoptosis, characterized as a prominent form of programmed cell death, holds significance in its connection with NS [21]. In 2001, caspase-1-dependent pro-inflammatory programmed cell death was defined as pyroptosis [22]. The activation of the NLRP3 inflammasome triggers pyroptosis. The NLRP3 inflammasome comprises NLRP3, an apoptosis-associated speck-like protein containing a caspase recruitment domain, and pro-caspase-1. Its assembly is triggered by the binding of various pathogen-associated molecular patterns or DAMPs to pattern recognition receptors [23]. The NLRP3, apoptosis-associated speck-like protein containing a caspase recruitment domain, and pro-caspase-1 complex activates caspase-1, culminating in the maturation of pro-inflammatory cytokines IL-1β and IL-18. At the end of the NLRP3 inflammasome cascade, GSDMD forms the membrane pore of the cell surface and drives cell swelling, bursting and programmed death, releasing pro-inflammatory cytokines (called ‘pyroptosis’) [7].

The NLRP3 inflammasome and pyroptosis cascade have been investigated in various kidney diseases, including chronic kidney disease [24], diabetic nephropathy [25] and lupus nephritis [26]. Ox-LDL is a DAMP that can activate the NLRP3 inflammasome [27]. Severe dyslipidaemia occurs frequently in patients with MCD. Although there are some reports describing the elevation of serum ox-LDL in NS [28, 29], a detailed analysis of urine ox-LDL has not previously been conducted. We emphasize the significance of assessing urinary ox-LDL as podocytopathy plays a crucial role in MCD, and podocytes within the glomerular tuft experience continuous exposure to urine. In our study, urine ox-LDL levels were significantly elevated in patients with MCD. These results encouraged us to investigate the activation of NLRP3 and the pyroptotic pathway in MCD. As the first step in assessing this pathway, we performed double immunofluorescence staining for the podocyte markers p57, NLRP3 and GSDMD. The results showed that NLRP3-positive podocytes significantly increased in patients with MCD.

In contrast, GSDMD^+^ podocytes were barely detected in the control and MCD groups. GSDMD activation is the final step of the pyroptosis cascade [30], which may be the reason for the rarity of GSDMD^+^ podocytes in the glomerular tufts. Next, we examined urinary IL-18 levels using ELISA. We analysed urinary IL-18 levels, as IL-18 can be released from cells undergoing pyroptosis. We hypothesized that podocyte pyroptosis might impact the levels of IL-18 in urine. As expected, urinary IL-18 levels were significantly higher in patients with MCD than in healthy controls. Immunohistochemical staining for IL-18 suggested that IL-18^+^ podocytes in the tuft were limited in both the control and MCD groups; this suggests the possibility that the podocytes undergoing pyroptosis might be promptly drained and exfoliated into the urine.

CD36 is a scavenger of ox-LDL [10]. In the kidneys, podocytes and tubular cells predominantly express CD36 [12]. The relationship between CD36 and various renal diseases, such as chronic kidney disease [31], diabetic nephropathy [25] and lupus nephritis [32], has been reported in the literature. In our previous study, we observed a correlation between the upregulation of CD36 and the activation of NLRP3/pyroptosis in injured murine podocytes [13]. Furthermore, we demonstrated a correlation between CD36 expression and the extent of podocyte injury and proteinuria. In the present study, double immunofluorescence staining for p57 and CD36 revealed an increase in CD36 expression in podocytes in cases of MCD compared with controls.

These results suggested that the NLRP3 inflammasome was induced through ox-LDL influx via activated CD36 in the podocytes of patients with MCD. Yang *et al*. reported that CD36 and NLRP3 inflammasome activation was induced in a mouse NS model [33], which strongly supports our hypothesis. In this study, urinary IL-18 levels were significantly reduced by steroid therapy but did not drastically affect the expression of CD36 and NLRP3 in podocytes. We considered that urinary ox-LDL levels decreased in patients receiving steroid therapy, which might regulate pyroptosis in MCD and result in the reduction of urinary IL-18 levels. In this study, we focused solely on the ox-LDL levels. However, there are several candidates for the ligand of CD36, such as long-chain free fatty acid, oxidized phospholipids and apolipoprotein C3, which might have affected the results of this study [34, 35]. The effects of steroid therapy on the NLRP3 inflammasome/pyroptosis cascade have not been fully elucidated in nephrology. Richard *et al*. reported that the NLRP3 inflammasome is related to the progression of severe steroid-resistant asthma, and direct therapy for the NLRP3 inflammasome could have therapeutic potency [36], which suggests the possibility that steroid therapy alone could not regulate the NLRP3 inflammasome perfectly. For decades, steroid therapy has been the main treatment for MCD; however, severe side effects are an existing problem. Therefore, a therapeutic strategy targeting the NLRP3 inflammasome could be an option for treating NS, including MCD.

This study had several limitations. First, the number of patients was limited; thus, the clinical backgrounds of the patients could not be statistically aligned. Secondly, the inclusion of only Japanese patients may limit the generalizability of the findings. Thirdly, kidney sections from kidney donors were used as controls for histological experiments; it is important to note that renal transplantation is an invasive procedure for kidney cells, introducing a potential influence on the obtained results.

In conclusion, our results demonstrate that there is a possibility that NLRP3 inflammasome and pyroptosis cascade might contribute to the pathogenesis or progression of MCD. In the current study, elevated urine ox-LDL levels were observed along with CD36/NLRP3 activation. While these findings imply the induction of pyroptosis in MCD, the exploration of pyroptosis in podocytes remains challenging. Therefore, additional investigations are needed to delve deeper into this aspect.

## Supplementary Material

sfae216_Supplemental_File

## Data Availability

The research data will be shared if the corresponding author considers the request necessary and reasonable.
