# Supplementary material for: Activation of the inflammasome and pyroptosis cascade in podocytes of patients with minimal change disease
Source: Clin Kidney J. 2024 Jul 16;17(8):sfae216. doi: 10.1093/ckj/sfae216 (PMC11304592; doi:10.1093/ckj/sfae216)

Supplemental Material

Figure legends

Supplemental Figure 1. Overview images of immunofluorescence staining for p57 (green), NLRP3 (red), and DAPI (blue).

Immunofluorescence staining for p57+ podocytes showed NLRP3 expression in patients with MCD when compared to those in kidney donors. Representative images of control (A) and MCD (B) groups. Magnification: ×100 (A and B) and ×400 (A' and B'). Bar: 200 µm. Dotted square: Representative part of the image (shown in A' and B').

Abbreviations: DAPI, 4′,6-diamidino-2-phenylindole; MCD, minimal change disease; NLRP3, NOD-like receptor thermal protein domain-associated protein 3.

Supplemental Figure 2. Overview images of immunohistochemical staining for IL-18.

Immunohistochemical staining for IL-18 showed that IL-18+ cells were barely detectable in control kidney donors or patients with MCD. Representative images of control (A) and MCD (B) groups. Magnification: ×100 (A and B) and ×400 (A' and B'). Bar: 200 µm. Dotted square: Representative part of the image (shown in A' and B').

Abbreviations: IL, interleukin, MCD, minimal change in disease.

Supplemental Figure 1

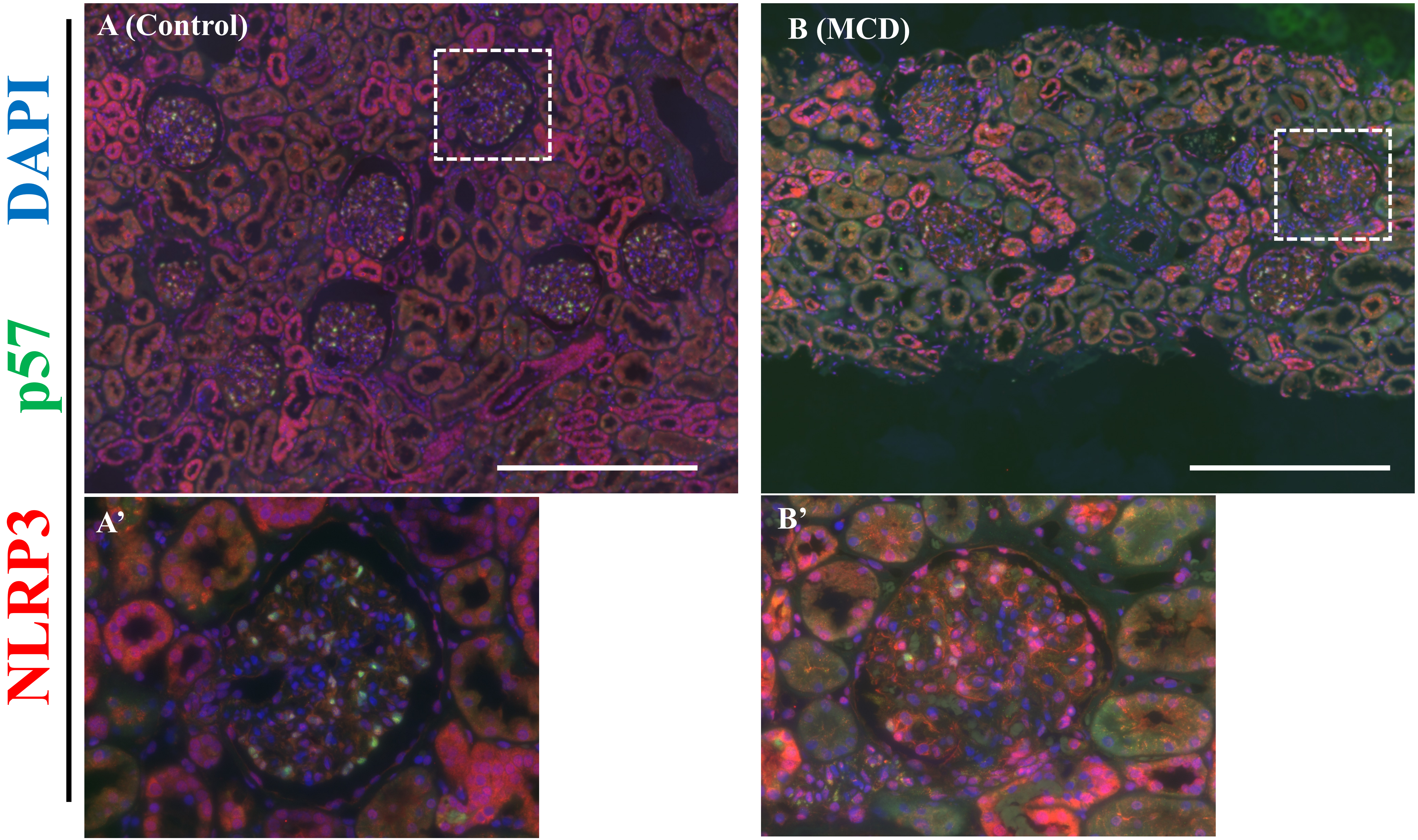

Supplemental Figure 2

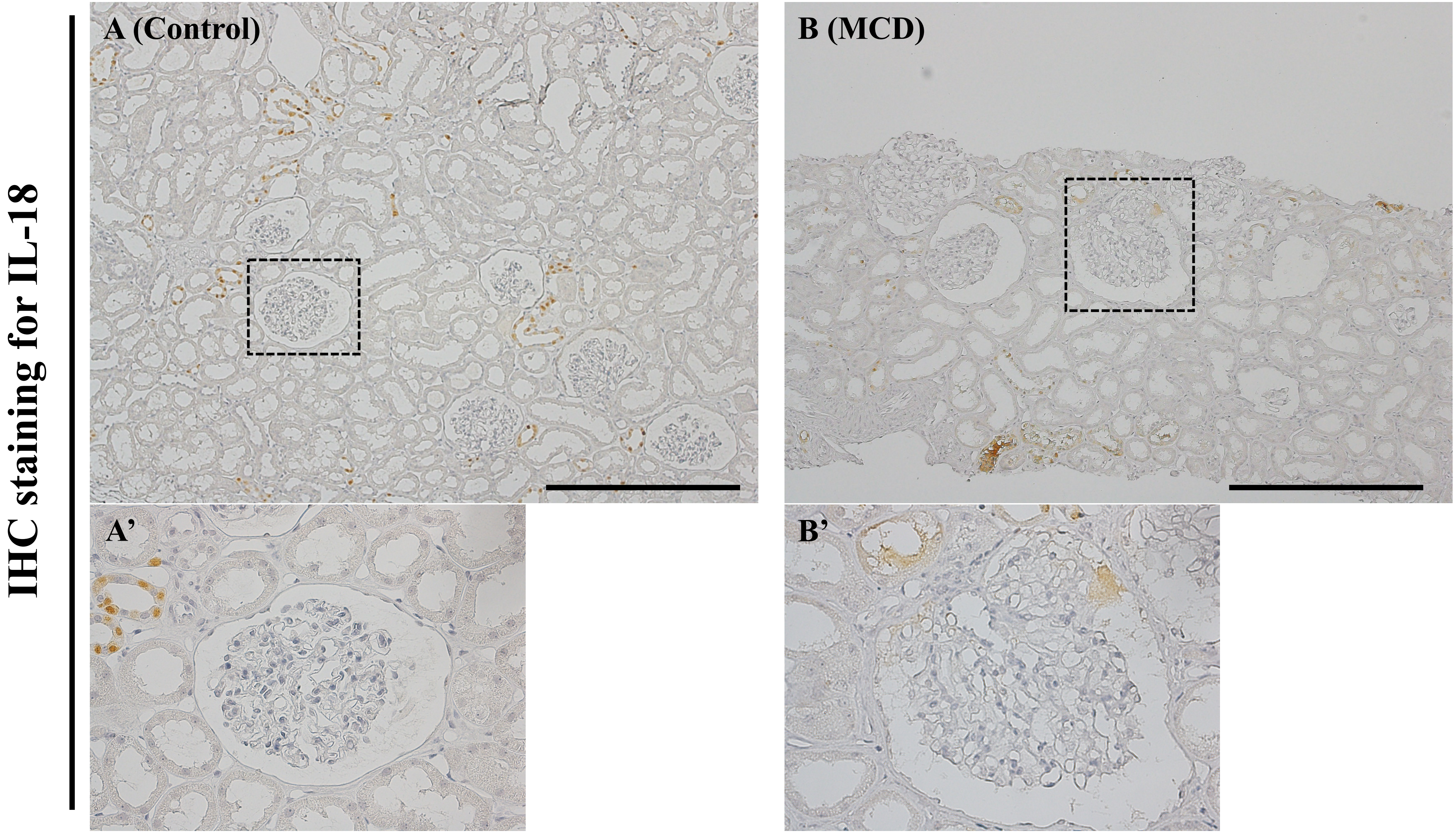

Supplement: sfae216_Supplemental_File [file sfae216_supplemental_file.pdf]
